# Lung Microbiome in Cystic Fibrosis

**DOI:** 10.3390/life11020094

**Published:** 2021-01-27

**Authors:** Filippo Scialo, Felice Amato, Gustavo Cernera, Monica Gelzo, Federica Zarrilli, Marika Comegna, Lucio Pastore, Andrea Bianco, Giuseppe Castaldo

**Affiliations:** 1 Dipartimento di Scienze Mediche Traslazionali, University of Campania “L. Vanvitelli”, 80131 Napoli, Italy; andrea.bianco@unicampania.it; 2 CEINGE, Biotecnologie Avanzate, 80145 Napoli, Italy; felice.amato@unina.it (F.A.); gustavo.cernera@unina.it (G.C.); monica.gelzo@unina.it (M.G.); federica.zarrilli@unina.it (F.Z.); marika.comegna@unina.it (M.C.); lucio.pastore@unina.it (L.P.); giuseppe.castaldo@unina.it (G.C.); 3 Dipartimento di Medicina Molecolare e Biotecnologie Mediche, Università di Napoli Federico II, 80145 Napoli, Italy

**Keywords:** cystic fibrosis, lung, microbiome, CFTR

## Abstract

The defective mucociliary clearance due to CFTR malfunctioning causes predisposition to the colonization of pathogens responsible for the recurrent inflammation and rapid deterioration of lung function in patients with cystic fibrosis (CF). This has also a profound effect on the lung microbiome composition, causing a progressive reduction in its diversity, which has become a common characteristic of patients affected by CF. Although we know that the lung microbiome plays an essential role in maintaining lung physiology, our comprehension of how the microbial components interact with each other and the lung, as well as how these interactions change during the disease’s course, is still at an early stage. Many challenges exist and many questions still to be answered, but there is no doubt that manipulation of the lung microbiome could help to develop better therapies for people affected by CF.

## 1. Introduction

In the last two decades, the development of new technologies, such as next-generation sequencing (NGS), has completely changed the idea that lungs are sterile organs that are infected with pathogens only during diseases. Nowadays, we know that healthy lungs are populated by intricate and diverse communities, such as bacteria, fungi, viruses, and archaea, which form the microbiome [1]. 

Since our knowledge about the role in lung physiology of the fungi, viruses, and archaea is not as advanced as it is for bacteria, in this review, with the term “microbiome”, we mainly refer to its bacterial component. Bacteria can be pathogenic, causing/exacerbating disease conditions, or commensal, exerting beneficial functions for the host. In fact, it has been demonstrated that bacteria have many important roles in maintaining lung physiology [2]; furthermore, they can interact with one another and with the cellular environment by affecting gene expression, protein/enzyme function, metabolism, and immunity [3,4].

In addition, the evidence that a decrease in microbial diversity is associated with the severity of pulmonary pathologies has made the lung microbiome an attractive target in the search for new therapies [5]. One of the pulmonary pathologies for which lung microbial diversity has a great impact is cystic fibrosis (CF) [6].

CF is a multisystemic disease caused by a defect in the production, folding, or function of the CF transmembrane conductance regulator (CFTR) protein [7]. It exerts a role as a chloride ion channel and has the important function of maintaining the water–salt balance; it is thus mainly involved in the transport of chloride, but also of other ions, such as sodium and bicarbonate. CFTR is expressed in both epithelial and non-epithelial cells of different organs, such as the lungs, pancreas, intestine, liver, reproductive organs, sweat glands, heart, kidney, and nervous system [8]. Although a malfunctioning CFTR gives rise to multisystemic diseases, the major cause of death in CF patients is the obstructive pulmonary condition that arises from a vicious cycle of infections and acute inflammation, leading to lung tissue damage and reduced airway exchange [8]. From their first months, the lungs of CF patients are, in fact, colonized by pathogens such as *Pseudomonas aeruginosa* (*P. aeruginosa*), whose chronic colonization is also due to the presence of polymorphisms in modifier genes that modulate ciliary beat frequency, promote the production of NO, and stimulate the release of immune peptides [9,10]. This makes antibiotic treatment inevitable; although necessary, it contributes to the development of dysbiosis, an unbalanced microbial diversity that selects for a specific bacteria taxon, causing the long-term reduction of lung function. Furthermore, it is well known that CF severity varies greatly among subjects, even in siblings that carry the same CFTR mutations [11]. A different lung microbiome composition could be the key to explaining the heterogeneity of the clinical manifestation, which would help to achieve a better comprehension of this pathology.

Therefore, the lung microbiome represents an attractive target for developing new patient-based CF therapies and diagnostic tools that can help to monitor the disease stage and be used by clinicians to predict exacerbations. The comprehension of the lung microbiome’s composition and its function in pathologies could also be pivotal in understanding other pulmonary pathologies where the efforts to find better ways to assess and diagnose disease progression are still at the center of intense research [12,13,14]. In this review, we will summarize key information about the lung microbiome in CF and ask what we think are some important questions that the future CF research field should address.

### Molecular and Cellular Characteristics of Cystic Fibrosis

The lower airways are constantly exposed to pathogens, pollution, and toxic matter. Therefore, the mechanisms of surveillance and protection have a key role in preserving lung function. Normally, the epithelial cells lining the conducting airways are protected from pathogens by the airway surface liquid (ASL). This is composed of a phase of fluid and water that allows the beating of cilia located at the apical part of the epithelial cells and an upper mucus layer where pathogens get trapped. The constant cilia movement is crucial for removing the trapped pathogens and avoiding unwanted bacterial colonization. A tightly controlled ion and water exchange across epithelial cells is needed to maintain the correct hydration of the ASL. This is achieved through the integrated functions of different channels, such as ENaC (Epithelial sodium channel) and CFTR, which are able to absorb sodium (Na^+^) and secrete chloride (Cl^−^), respectively. Previous studies have demonstrated that a functional CFTR is necessary to regulate and maintain ASL hydration [15,16]. In CF subjects, the impaired Cl^−^ secretion depletes the ASL, causing an accumulation of mucus and creating the perfect conditions for pathogen colonization. This also has a huge impact on the activity of antibacterial proteins that reside in the ASL. SPLUNC1, which is part of the bactericidal permeability-increasing (BPI) protein family, has been shown to exert antimicrobial activity against *P. aeruginosa* and *Burkholderia*, and is inactivated by the acidic environment formed in the lungs of CF subjects [17]. CFTR is also involved in the transport of glutathione (GSH) in the ASL, a well-known antioxidant molecule. Although reactive oxygen species (ROS) play an important role in fighting pathogen colonization in the ASL, it is well documented that an excess of ROS can induce damage and trigger inflammation. Therefore, in CF subjects, malfunctioning CFTR produces a reduction in the ASL with a consequent accumulation of mucus, which favors bacterial colonization. Moreover, the acidic pH and the inability to transport GSH inactivate antimicrobial proteins and cause the imbalance in the antioxidant potential of the ASL, thus producing epithelial damage.

## 2. How Does the Lung Microbiome in CF Patients Change with Age and Disease Stage?

From their first months of life, CF infants have a different lung microbiome (LM) composition from that of healthy subjects [18], although many studies have demonstrated that in the first decade of life, the lungs of CF subjects still preserve some diversity, as they are colonized with non-typical CF pathogens. The genera that are normally found are Streptococcus, Prevotella, Rothia, Veillonella, Gemella, Neissera, Actynomyces, and Haemophilus [18,19]. Interestingly, the microbiome composition of bronchoalveolar lavage fluid (BALF) from CF patients in the first year of life does not seem to be identical with the one present in oropharyngeal swabs or nasopharyngeal samples [18]. This has important clinical implications, since, at least in infants, it makes the upper respiratory tract niches inadequate for the determination of lung microbial diversity and, most importantly, for the identification of all pathogens. Furthermore, especially under disease conditions, the microbiome could be different between the left and right lungs [20] and in the different areas of the thick mucus layers characteristic of the CF patients [21], reinforcing the idea that analyzing the microbiomes from patients’ BALFs would make diagnosis and therapies more accurate. As described earlier, in young CF patients, the microbiome still seems to preserve some diversity, with *Streptococcus* being the predominant taxon; the common CF pathogens (*Staphylococcus* and *P. aeruginosa*) comprise about 50% of the total microbial community. Starting from the second decade of life, microbial diversity begin to decrease, which is probably also due to the heavy antibiotic use, and the common CF pathogens, such as *P. aeruginosa* and *Burkholderia*, become more abundant, thus decreasing microbiome diversity [19]. Their presence is also correlated with the disease’s severity. Lung function measured by FEV (forced expiratory volume) decreases with age and correlates with less microbiome diversity and dominance of these two pathogens, which, when present, decrease lung function in young CF subjects as well [19]. Another important aspect to consider is the episodes of exacerbation with an increase in respiratory symptoms, which are very common and persistent during the lives of CF patients [22]. The most logical explanation would be that these episodes are due to an increase in CF pathogens, causing a consequent reduction in microbial diversity. Unfortunately, there is no consensus in the literature about this matter, with some clinical reports demonstrating a change in diversity during exacerbation [23,24,25], while others do not report any variation in microbial composition [26,27]. Although this discordance makes our understanding of host–pathogen interaction during exacerbations more complicated, it opens new conceptual views, where different CF subjects could manifest exacerbations due to: (i) an increase in the amount of a specific pathogen; (ii) a switch in the metabolic activity of a specific bacterial family; (iii) some anaerobic bacteria that are not normally associated with the disease state that can act as pathogens under particular conditions [28]. Therefore, although a late diagnosis of CF is rare nowadays [29], an early assessment of the disease in association with lung microbiome composition could reduce the occurrence of complications later in life.

## 3. How Do CF Therapies Affect the Lung Microbiome?

Although the enormous progress in the development of new therapies has substantially increased the quality of life and life expectancy of CF patients, antibiotic treatment is still the first line of defense against recurrent infections in CF patients. It is used especially to treat episodes of exacerbation, which, as we described above, are frequent during the lifetimes of CF subjects [30]. Many studies have demonstrated that, although necessary, antibiotic therapies are the first cause of a reduction in microbial diversity [31,32]. CF patients are subject to continuous use of different antibiotics and can develop what is called multidrug resistance (MDR), which can help to select for a specific taxon, thus further decreasing microbial diversity [33]. Another important aspect to note is the untargeted action that antibiotics can have on non-pathogenic bacteria, applying additional pressure on the lung microbial community [34]. Furthermore, it has been shown that antibiotic therapies used to eradicate *P. aeruginosa* can make the conditions favorable for acquiring *Aspergillus* in young CF subjects [35], causing a further imbalance in microbiome composition.

In the last decade, new therapies have been developed, changing from the classical treatment of the downstream symptoms to the development of small drugs named potentiators and correctors, which can improve the CFTR processing or function in patients with some classes of CFTR mutations [36]. It is interesting to note that, alone or in combination, these drugs exert antimicrobial activity both in vitro and in vivo. For instance, Ivacaftor has been demonstrated to reduce the viability and growth of *P. aeruginosa*, *Streptococcus pneumoniae*, and *Staphilococcus aureus*, and its activity was potentiated in the presence of antibiotics [37]. Similar results were obtained in two other independent studies, where Ivacaftor alone or in combination with ciprofloxacin showed the same antimicrobial activity against the same pathogens [38,39]. Interestingly, the assessment of microbiome changes in CF subjects treated with Ivacaftor also showed an initial reduction in *P. aeruginosa* colonization, but did not achieve a complete eradication, with the *P. aeruginosa* level increasing after one year of treatment [40,41].

Understanding how and if these CFTR modulators directly affect the microbiome in CF patients has many clinical implications. For instance, if the correction of CFTR function would restore microbiome diversity and decrease pathogen loads, then assessing the microbiome composition could be used as a screening tool to follow disease severity and progression. If this would not be the case, then we would know that restoring CFTR function is not enough, and re-establishing the microbiome diversity could represent the key for restoring lung function. Furthermore, if pathogen loads would not decrease, antibiotic treatment in combination with these drugs would still be necessary.

## 4. Can We Improve Lung Function by Restoring Microbiome Diversity?

As described earlier, a decrease in microbial diversity is intimately linked with a deterioration in lung function in CF [19]. This has opened the way to the interesting hypothesis that restoring its diversity would have a beneficial effect on lung physiology, consequently improving its function. Although, at a first glance, this could appear to be a simple task, differently from the gut microbiome, considerable challenges exist in manipulating and following the changes in the lung microbiome in patients with CF. Firstly, lungs have a more complex anatomical structure than the gut [42]. Furthermore, collecting fecal samples to study the gut microbiome is not invasive and could be repeated at any time to monitor its dynamic changes. This is different for the lungs. While BALF would give a very close picture of the lung microbiome, differently from oropharyngeal or nasopharyngeal samples [18], it is an invasive procedure and cannot be performed many times in CF patients that are more prone to infection and in which the lungs could be already damaged. This greatly limits our possibilities for studying if changes in lung microbial composition correlate with lung function or if new therapies can repristinate its diversity. Many studies have, in fact, successfully manipulated the gut microbiome by studying its impact on pulmonary pathology, unraveling a gut–lung axis [43,44,45], while very few studies have tried to manipulate the lung microbiome, leaving its gut counterpart unaltered. For instance, in a proof-of-concept study, Le Noci and colleagues successfully used probiotic aerosol therapy to demonstrate its immunomodulatory effect in a mouse model of lung cancer [46], and it would be interesting to see if the same approach could be used as a combination therapy with CFTR modulators.

## 5. How Should We Look at the Microbiome to Understand Its Complexity?

**Figure 1:**
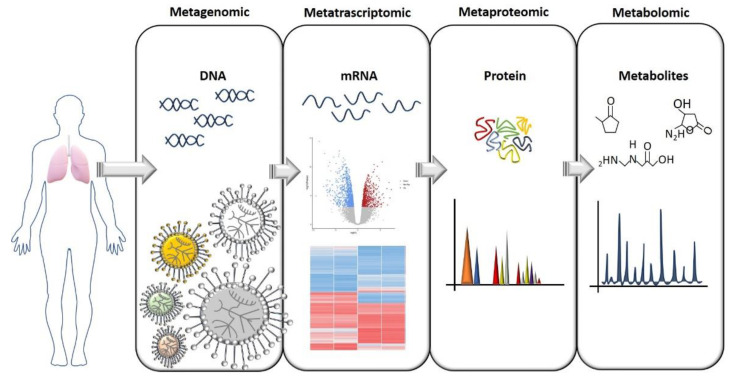
A flow chart for study of the microbiome. The OMICS approaches needed to gain a full comprehension of the microbiome’s role both in heath and pathologies are illustrated here. The identification of a specific bacterial composition in pathological conditions should ideally be followed by an assessment of gene and protein expression, and especially an assessment of which metabolites are produced that can influence tissue homeostasis.

To understand how the lung microbiome could be used to monitor CF development, predict exacerbation, and function as an ally for developing better therapies, we need to understand its complexity and what kinds of interaction microbial communities have within the lung. This means that we must look at the microbiome in a different way, and not only ask “What is there?” and “How much is there?”. The answers to these questions are, of course, an important part of the picture, but now we know that they are just the beginning. As previously anticipated, in CF, reduced diversity is linked to lung function, but what exactly does “reduced diversity” mean when we try to link it with lung function? Which physiological functions were those bacteria responsible for that are now reduced or missing? How do these bacteria communicate to maintain a healthy lung environment? What is the proportion that each bacterial family needs to maintain in the community before becoming pathogenic? As hypothesized earlier, CF exacerbations could be due to a switch in the metabolic activity of specific bacteria families that were previously inactive or to a family of non-pathogenic bacteria that, in a specific situation, can behave as a pathogen, and not due to a change in microbiome composition [26,28]. Therefore, microbiome composition studies should be followed by: (i) metagenomic analysis to assess the functional capacity of the microbiome, (ii) meta-transcriptomic analysis to assess gene expression, (iii) meta-proteomics to study the catalytic functions, and (iv) metabolomics to study the metabolic activity (Figure 1). These are the necessary steps to put in place to fully understand the microbiome’s complexity and the interactions within the lung during the disease course. Particular attention should be given to the study of the microbiome’s metabolic activity. We know that bacteria, through the production of specific metabolites, can communicate with each other and with the host organ, and can even influence molecular pathways in distal tissue [47]. Therefore, the identification of metabolites and how the metabolic activity changes during disease progression has become a piece of crucial information that can potentially lead to the identification of biomarkers. This would have a huge impact on clinical practice. In fact, although microbiome studies started more than a decade ago, understanding the large amount of data obtained from OMICS analysis and translating them into a clear message that is able to inform and guide treatments are probably the biggest challenges that the microbiome field needs to overcome.

## 6. Discussion

This is an exciting time for the CF field. The development of new molecular techniques and new classes of drugs that are able to correct or potentiate CFTR folding and/or function is revolutionizing the therapies and lives of many people affected by this pathology. Although many steps towards a cure have been taken, we are still far from the full comprehension of this multisystemic disease. In the last decade, there has been a growing body of evidence demonstrating that lung microbiome diversity plays a key role in maintaining lung physiology, and its dysregulation can lead to chronic lung diseases. In CF patients, lung microbiome dysbiosis is already evident in the first years of life, and although the restoration of CFTR function with a combination of correctors and potentiators is proving to be the right path to follow, it might not be enough if a healthy/balanced microbiome is not restored. Unfortunately, due to technical difficulties in sample collections, costs, and data analysis, we are still not able to use the precious information that the microbiome studies harness, which could better inform clinical practices.

However, we have already changed how we look at the microbiome, and we understand that examining a single bacterial family cannot allow us to reveal the intricate world that is a microbial system and how it communicates with us. We are confident that future technological innovations will advance our comprehension of the role that the microbiome plays in CF, giving patients the opportunity to have better therapies and new diagnostic tools.
